# A Minimal Model Framework for Robust CAR-T Cell and Oncolytic Virus Combination Therapy

**DOI:** 10.21203/rs.3.rs-8680401/v1

**Published:** 2026-01-27

**Authors:** Aisha Tursynkozha, Yang Kuang

**Affiliations:** 1 School of Artificial Intelligence and Data Science, Astana IT University, Astana, 010000, Kazakhstan.; 2 School of Mathematical and Statistical Sciences, Arizona State University, Tempe, AZ, 85287, USA.

**Keywords:** Glioblastoma, CAR-T therapy, Oncolytic virus, Combination therapy, Therapeutic synergy, Mathematical modeling

## Abstract

Glioblastoma remains one of the most lethal brain cancers. Combination therapy using CAR-T cells and oncolytic viruses shows promise, yet mechanisms underlying synergy remain poorly understood. We develop mathematical models to analyze IL-13R***α***2-targeting CAR-T cells and the oncolytic virus C134 using patient-derived glioblastoma data. We present a minimal model framework for predicting combination immunotherapy outcomes. Applying timescale separation between rapid viral and slower cellular dynamics, we derive quasi-steady-state (QSS) approximations that reduce complexity while maintaining accuracy. The QSS model uses 9 parameters compared with 11 in the full model and achieves comparable fits. Model comparisons using the Akaike Information Criterion indicate that the QSS model is generally favored; it consistently yields lower AIC values for oncolytic virus monotherapy and produces lower AIC values in three of four combination therapy conditions. Models with and without CAR-T exhaustion produce identical fits, indicating that exhaustion dynamics do not improve predictions within the 72-hour observation window. Overall, our results demonstrate that simplified QSS formulations effectively capture viral dynamics and provide a practical framework for optimizing combination immunotherapies.

## 1 Introduction

Glioblastoma multiforme (GBM) is the most common and aggressive brain tumor in adults, characterized by rapid growth, diffuse infiltration, and high recurrence rates despite standard treatments such as surgical resection, radiotherapy, and temozolomide chemotherapy [1, 2]. Survival improvements over recent decades have been limited, reflecting the highly heterogeneous and treatment-resistant nature of GBM [3]. These challenges have motivated the development of diverse therapeutic strategies, including immunotherapies and locoregional approaches, aimed at eliminating tumor cells, stimulating anti-tumor immunity, and overcoming barriers such as the blood-brain barrier and intrinsic resistance [4].

Among these emerging approaches, immunotherapies have attracted particular attention, with Chimeric Antigen Receptor (CAR) T-cell therapy emerging as a promising strategy. CAR-T therapy involves engineering a patient’s T cells to recognize and selectively eliminate tumor cells [5]. In the context of GBM, CAR-T cells targeting IL-13R*α*2 have demonstrated tumor regression [6]. However, their effectiveness in solid tumors is still limited by poor T-cell infiltration, an immunosuppressive tumor microenvironment, and antigen heterogeneity [7].

To address some of these limitations, oncolytic virus (OV) therapy has been explored as a complementary strategy. The concept of using viruses to treat cancer dates back to early 20th-century observations, when viral infections were occasionally associated with spontaneous tumor regression [8]. Modern OVs exploit this phenomenon through a dual mechanism: selective infection and lysis of malignant cells, coupled with stimulation of anti-tumor immune responses. Viral replication within tumor cells triggers cell death and the release of new virions, which spread to neighboring cancer cells. Importantly, this process induces immunogenic cell death, releasing tumor antigens and danger signals that activate the immune system and convert immunologically “cold” tumors into “hot” ones. When combined with chemotherapy or other immunotherapies, OV therapy can achieve synergistic effects, enhancing efficacy while minimizing toxicity [9–11].

Since CAR-T and OV therapies target tumors via distinct but complementary mechanisms, their combination has emerged as a promising strategy. OVs can remodel the tumor microenvironment to support CAR-T function by reducing immunosuppression, enhancing antigen presentation, and promoting chemokine-mediated recruitment of effector cells [12]. Conversely, CAR-T cells can target virus-infected tumor cells, potentially increasing viral spread and overall treatment efficacy. Understanding how these therapies interact and optimizing their combination requires quantitative frameworks that can capture the underlying dynamics.

Mathematical modeling has proven to be a valuable tool for understanding the dynamics and predicting outcomes of complex cancer immunotherapies [13–15]. Mechanistic ordinary differential equation (ODE) models of CAR-T cell therapy for glioma have, for instance, investigated how multiple CAR-T cells bind to individual tumor cells and how parameters such as expansion rates and antigen receptor densities influence treatment success and tumor elimination kinetics [16]. Adhikarla et al. [17] employed ODE modeling to optimize the scheduling of CAR-T therapy in combination with targeted radionuclide therapy, showing that the timing and sequencing of treatments critically affect therapeutic outcomes. Similarly, Conte et al. [18] introduced a kinetic-theory–based approach to tumor–immune modeling, deriving macroscopic dynamics from microscopic cellular interactions and highlighting how spatial structure and immune cell state transitions shape tumor-immune behavior. Mahasa et al. [27] investigated dosing strategies for CAR-T and OV combination therapy, revealing that virus-induced synergism can prevent tumor elimination and that simultaneous administration induces more sustained tumor cell reduction compared to sequential treatment. More recently, Pell [19] developed an ODE framework demonstrating that immunostimulants such as NHS-muIL12 can both boost immune activity and induce tumor PD-L1 upregulation, thereby reducing the efficacy of PD-L1 checkpoint blockade and explaining treatment failure. Building on these foundations, Conte et al. [20] developed an early data-driven combination-treatment model integrating uninfected and infected tumor cells, viral replication, and CAR-T dynamics in the context of GBM. Inspired by this approach and the foundational work of Pell et al. [19], we propose an alternative minimal model for analyzing CAR-T cell and oncolytic virus combination therapy. The model tracks four key populations: uninfected and infected tumor cells, viral particles, and CAR-T cells, including explicit dynamics for CAR-T cell exhaustion. To examine the balance between biological detail and model tractability, we also consider a reduced formulation in which exhaustion effects are represented implicitly through modified CAR-T cell death rates over a 72-hour timescale. In addition, we apply timescale separation to distinguish fast viral dynamics from the slower evolution of cellular populations, allowing for quasi-steady-state approximations.

The model is calibrated to experimental data from Conte et al. [20] across multiple effect-or-to-target ratios and multiplicities of infection. Using this framework, we analyze interactions between CAR-T and oncolytic virus therapies, evaluate the impact of model simplifications, and identify mechanisms that are essential for reproducing key experimental trends relevant to combination treatment.

Sections 2 and 3 describe the experimental data, present the full mathematical model, introduce reduced and quasi-steady-state formulations, and apply these models to combination therapy and monotherapy scenarios using data fitting. Section 4 presents quantitative model comparisons across formulations, including performance metrics and identifiability analysis. Section 5 discusses biological interpretation and implications for therapeutic optimization.

## 2 Main Model

### 2.1 Experimental Data

The experimental data used to validate our mathematical model are obtained from recent investigations by Conte et al., who systematically examined combination therapy involving oncolytic virus C134 and IL-13R*α*2-targeting CAR-T cells against patient-derived glioblastoma cultures [20]. C134 is a herpes simplex virus (HSV)-based oncolytic virus that selectively infects and lyses tumor cells, utilizing cancer-specific defects in antiviral defenses. Upon infection, C134 replicates within tumor cells, triggering cell lysis and releasing new viral particles for subsequent infection cycles. The CAR-T cells were engineered to target IL-13R*α*2, a receptor overexpressed in glioblastomas but with limited expression in normal brain tissue, thereby enabling selective recognition and elimination of tumor cells.

Experiments employed PBT030 primary glioblastoma cultures with high IL-13R*α*2 expression. Cells were plated at 2 × 10^4^ per well with three biological replicates (n=3) per condition. Cultures were initially infected with C134 at multiplicities of infection (MOI=0.03,0.02,or0.008), representing different viral particle-to-cell ratios. Two hours later, CAR-T cells were introduced at effector-to-target (E:T) ratios of 1:10, 1:25, or 1:50, corresponding to 2000, 800, and 400 cells per well, allowing the virus to establish infection prior to CAR-T cell engagement.

Tumor dynamics were monitored using the xCELLigence system, which continuously measures electrical impedance as cells adhere to electrode-coated surfaces. The resulting Cell Index (CI), a dimensionless proxy for viable cell counts, was recorded every 15 minutes over 72 hours, yielding high-resolution temporal profiles (> 280 data points per condition). These measurements were used to estimate model parameters for both monotherapy and combination therapy.

### 2.2 Model Formulation

We develop a mathematical model to describe the tumor-immune-virus dynamics in the experiments described in Section 2.1. The model tracks four populations: uninfected tumor cells U(t), infected tumor cells I(t), oncolytic virus particles V(t), and CAR-T cells C(t). We begin with a detailed formulation incorporating the main biological mechanisms, then develop reduced models motivated by timescale separation. The dynamics of each population are determined by:

#### Uninfected Tumor Cells: U(t).

Uninfected tumor cells follow logistic growth dynamics, expressed as $\text{rU}\left(1-\frac{U+I}{K}\right)$, where r denotes the intrinsic growth rate and K represents the environmental carrying capacity accounting for space and nutrient limitations. The tumor population is reduced through two mechanisms: infection by the oncolytic virus, represented by $\frac{\beta \text{UV}}{U+I+a}$, where β denotes the infection rate coefficient and the saturation term U+I+a reflects crowding effects and limited receptor availability; and elimination by CAR-T cells through antigen recognition and cytotoxic mechanisms, represented by $\frac{d_{c}\text{CU}}{U+I+a}$, where $d_{c}$ denotes the killing rate coefficient. The unified saturation constant a represents a threshold reflecting the finite capacity of cellular interactions at high densities, accounting for both spatial crowding and shared metabolic constraints. The dynamics of uninfected tumor cells are therefore described by:

(1) $$\frac{\text{dU}}{dt}=\underset{\text{tumor}\;\text{proliferation}}{\underbrace{\text{rU}\left(1-\frac{U+I}{K}\right)}}-\underset{\text{virus}\;\text{infection}}{\underbrace{\frac{\beta \text{UV}}{U+I+a}}}-\underset{\text{CAR-T}\;\text{killing}}{\underbrace{\frac{d_{c}\text{CU}}{U+I+a}}}.$$

#### Infected Tumor Cells: I(t).

Infected tumor cells are generated when uninfected tumor cells become infected by the oncolytic virus at rate $\frac{\beta \text{UV}}{U+I+a}$. Once infected, tumor cells lose their proliferative capacity and do not undergo further division. The infected cell population is reduced through two processes: CAR-T mediated killing, represented by $\frac{d_{i}\text{CI}}{U+I+a}$, where $d_{i}$ denotes the killing rate coefficient; and viral lysis at rate $w_{I}$, which releases new viral particles into the tumor microenvironment. The dynamics of infected tumor cells are given by:

(2) $$\frac{\text{dI}}{\text{dt}}=\underset{\text{new}\;\text{infections}}{\underbrace{\frac{\beta \text{UV}}{U+I+a}}}-\underset{\text{CAR-T}\;\text{killing}}{\underbrace{\frac{d_{i}\text{CI}}{U+I+a}}}-\underset{\text{viral}\;\text{lysis}}{\underbrace{w_{I}I}}.$$

#### Oncolytic Virus: V(t).

Viral particles are produced upon lysis of infected tumor cells, with burst size b contributing to viral amplification. This production is represented by $bw_{I}I$. The viral population decreases through two mechanisms: consumption during infection of uninfected tumor cells at a rate $\frac{\beta \text{UV}}{U+I+a}$, and natural clearance through degradation and non-productive binding at a rate $w_{V}$. Combining these mechanisms, the oncolytic virus changes as:

(3) $$\frac{\text{dV}}{\text{dt}}=\underset{\text{virus}\;\text{production}}{\underbrace{bw_{I}I}}-\underset{\text{infection}\;\text{loss}}{\underbrace{\frac{\beta \text{UV}}{U+I+a}}}-\underset{\text{viral}\;\text{clearance}}{\underbrace{w_{V}V}}.$$

#### CAR-T Cells: C(t).

CAR-T cells expand in response to tumor antigen stimulation, modeled within a Michaelis-Menten framework as $\frac{\rho \text{CU}}{U+I+a}$, where ρ denotes the proliferation rate induced by uninfected tumor cells. The CAR-T population is subject to multiple loss mechanisms: natural apoptosis at a rate $w_{C}C$; and exhaustion due to prolonged antigen exposure, represented by $\frac{\varepsilon C(U+I)}{U+I+a}$, where ε denotes the exhaustion rate coefficient. The dynamics of CAR-T cells are represented by:

(4) $$\frac{\text{dC}}{\text{dt}}=\underset{\text{CAR-T}\;\text{expansion}}{\underbrace{\frac{\rho \text{CU}}{U+I+a}}}-\underset{\text{natural}\;\text{death}}{\underbrace{w_{C}C}}-\underset{\text{exhaustion}}{\underbrace{\frac{\varepsilon C(U+I)}{U+I+a}}}.$$

Collecting the above components, the complete model with CAR-T exhaustion is given by:

(5) $$\frac{\text{dU}}{\text{dt}}=\underset{\text{tumor}\;\text{proliferation}}{\underbrace{\text{rU}\left(1-\frac{U+I}{K}\right)}}-\underset{\text{virus}\;\text{infection}}{\underbrace{\frac{\beta \text{UV}}{U+I+a}}}-\underset{\text{CAR-T}\;\text{killing}}{\underbrace{\frac{d_{c}\text{CU}}{U+I+a}}},\;\frac{\text{dI}}{\text{dt}}=\underset{\text{new}\;\text{infections}}{\underbrace{\frac{\beta \text{UV}}{U+I+a}}}-\underset{\text{CAR-T}\;\text{killing}}{\underbrace{\frac{d_{i}\text{CI}}{U+I+a}}}-\underset{\text{viral}\;\text{lysis}}{\underbrace{w_{I}I}},\;\frac{\text{dV}}{\text{dt}}=\underset{\text{virus}\;\text{production}}{\underbrace{bw_{I}I}}-\underset{\text{infection}\;\text{loss}}{\underbrace{\frac{\beta \text{UV}}{U+I+a}}}-\underset{\text{viral}\;\text{clearance}}{\underbrace{w_{V}V}},\;\frac{\text{dC}}{\text{dt}}=\underset{\text{CAR-T}\;\text{expansion}}{\underbrace{\frac{\rho \text{CU}}{U+I+a}}}-\underset{\text{natural}\;\text{death}}{\underbrace{w_{C}C}}-\underset{\text{exhaustion}}{\underbrace{\frac{\varepsilon C\left(U+I\right)}{U+I+a}}}.$$

We summarize the model parameters with their biological interpretation and units in Table 1.

### 2.3 Parameter Estimation

We estimated model parameters by fitting predictions to experimental Cell Index measurements. The optimization employed the L-BFGS-B algorithm [21, 22] (limited-memory quasi-Newton method for bound-constrained nonlinear optimization) implemented in SciPy [23]. For each experimental condition and biological replicate, we minimized the Weighted Absolute Percentage Error (WAPE):

(6) $$\text{WAPE}=\frac{\sum_{i=1}^{n}\left|y_{i}-{\overset{ˆ}{y}}_{i}\right|}{\sum_{i=1}^{n}\left|y_{i}\right|}$$

where $y_{i}$ denotes the measured Cell Index and ${\overset{ˆ}{y}}_{i}$ the model prediction at time i.

Model performance was evaluated using three standard metrics: the Residual Sum of Squares (RSS), the adjusted $R^{2}$ coefficient, and the Akaike Information Criterion (AIC). The RSS, defined as

(7) $$\text{RSS}=\sum_{i=1}^{n}{\left(y_{i}-{\overset{ˆ}{y}}_{i}\right)}^{2},$$

represents the squared deviations between model predictions and experimental data. While WAPE was minimized during parameter fitting, RSS provides a standard measure of deviation, with lower values indicating better agreement between model and data.

The adjusted $R^{2}$ accounts for both the number of fitted parameters and the number of observations, and was calculated as

(8) $$R^{2}=1-\frac{\text{RSS}}{\text{TSS}},\;\text{TSS}=\sum_{i=1}^{n}{\left(y_{i}-\overset{‾}{y}\right)}^{2},\;R_{\text{adj}}^{2}=1-\frac{\left(1-R^{2}\right)(n-1)}{n-k-1},$$

where n is the number of observations, k the number of fitted parameters, and TSS the total sum of squares. A higher adjusted $R^{2}$ indicates a better model fit while accounting for model complexity.

The AIC penalizes model complexity relative to fit quality and was defined as

(9) AIC=nln(RSS/n)+2k.

A lower AIC indicates a better trade-off between goodness of fit and model simplicity.

## 3 Model Iterations and Comparisons

We use computational modeling to explore CAR-T cell and oncolytic virus treatment of glioblastoma and assess therapeutic synergy under combination therapy. We first analyze combination therapy, then monotherapies, and finally quasi-steady-state approximations for each scenario.

For each therapeutic condition, we present the governing equations and fit our model to experimental Cell Index measurements across all dosing conditions and biological replicates. Unless stated otherwise, all model parameters are estimated via nonlinear optimization, as detailed in Section 2.3. Baseline experimental conditions employed PBT030 glioblastoma cells, with therapeutic agents administered according to the protocols described in Section 2.1.

### 3.1 Combination Therapy Dynamics

#### 3.1.1 Full Model with CAR-T Exhaustion

The combination therapy case is described by model Eq. (5), which examines the effects of varying CAR-T cell dose (E:T ratios of 1:25 and 1:50) and viral load (MOI of 0.002 and 0.0008) on tumor dynamics. Tumor response is analyzed across four dose combinations, with three biological replicates per condition.

**Fig. 1:**
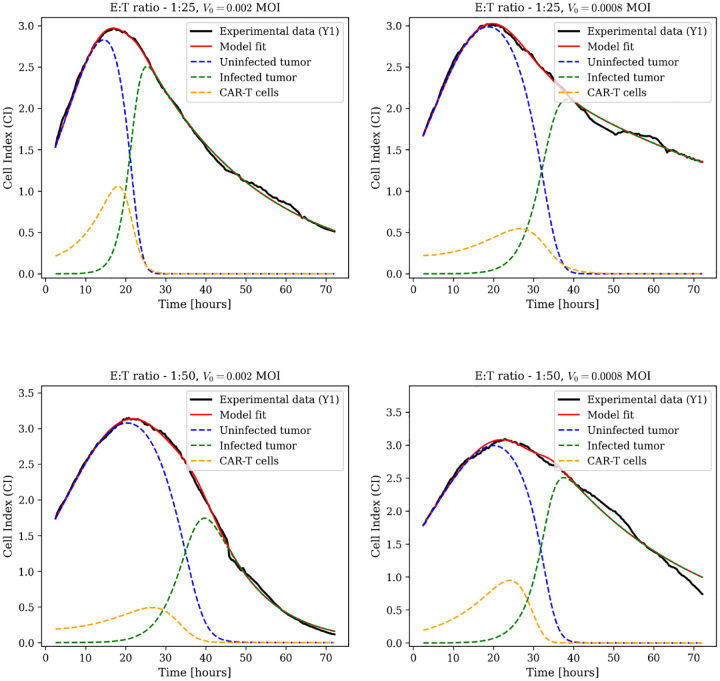
Combination therapy dynamics using the full model with CAR-T exhaustion. Model fits (red lines) to experimental Cell Index data (black lines) for biological replicate Y1 across different conditions: E:T ratio 1:25 with MOI 0.002, E:T ratio 1:25 with MOI 0.0008, E:T ratio 1:50 with MOI 0.002, and E:T ratio 1:50 with MOI 0.0008. Dashed blue lines represent uninfected tumor cell dynamics; dashed green lines show infected tumor cell dynamics; dashed yellow lines illustrate CAR-T cells. Additional replicates Y2 and Y3 are shown in Appendix A1.

Data-fitting results for biological replicate Y1 appear in Fig. 1, with additional replicates (Y2 and Y3) in Appendix A1. Experimental Cell Index measurements (black lines) are overlaid with model predictions (red lines). The model resolves associated population dynamics, including uninfected tumor cells (blue dashed), infected tumor cells (green dashed), and CAR-T cells (yellow dashed).

Sustained tumor suppression is achieved within 24 hours across all dose combinations and maintained throughout the 72-hour observation period. The dynamics highlight nonlinear interactions between viral oncolysis, CAR-T cytotoxicity, and exhaustion, particularly in how the two therapies differentially kill infected versus uninfected tumor cells. Parameter estimates are in Table A1.

#### 3.1.2 Reduced Model Without CAR-T Exhaustion

CAR-T exhaustion involves progressive transcriptional changes requiring sustained antigen exposure over days to weeks. Within the 72-hour experimental window, the primary loss mechanisms are direct cytotoxic killing and activation-induced cell death, both captured by the natural death term $w_{C}$. To test whether explicit exhaustion dynamics are necessary for accurate short-term predictions, we removed the exhaustion term −εC(U+I)/(U+I+a) from the CAR-T equation in Eq. 5:


$$\frac{\text{dU}}{\text{dt}}=\text{rU}\left(1-\frac{U+I}{K}\right)-\frac{\beta \text{UV}}{U+I+a}-\frac{d_{c}\text{CU}}{U+I+a},\;\frac{\text{dI}}{\text{dt}}=\frac{\beta \text{UV}}{U+I+a}-\frac{d_{i}\text{CI}}{U+I+a}-w_{I}I,\;\frac{\text{dV}}{\text{dt}}=bw_{I}I-\frac{\beta \text{UV}}{U+I+a}-w_{V}V,\;\frac{\text{dC}}{\text{dt}}=\frac{\rho \text{CU}}{U+I+a}-w_{C}C.$$

**Fig. 2:**
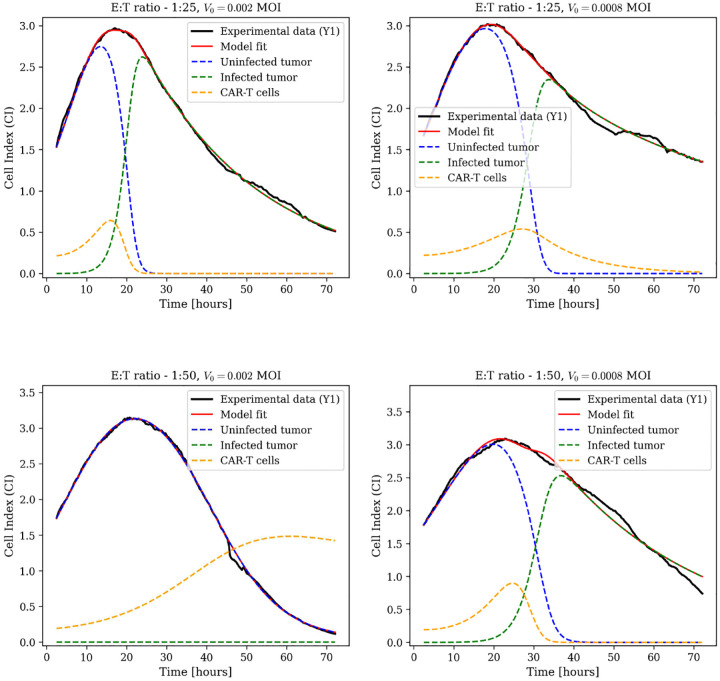
Combination therapy dynamics using the reduced model without CAR-T exhaustion. Model fits (red lines) to experimental Cell Index data (black lines) for biological replicate Y1 across different conditions: E:T ratio 1:25 with MOI 0.002, E:T ratio 1:25 with MOI 0.0008, E:T ratio 1:50 with MOI 0.002, and E:T ratio 1:50 with MOI 0.0008. Dashed blue lines represent uninfected tumor cell dynamics; dashed green lines show infected tumor cell dynamics; dashed yellow lines illustrate CAR-T cells. Additional replicates Y2 and Y3 are shown in Appendix A2.

Model fits for biological replicate Y1 across the same four dose combinations are shown in Fig. 2, with additional replicates (Y2 and Y3) in Appendix A2. Tumor suppression within 24 hours occurs across all conditions. The reduced model captures the same tumor suppression dynamics observed in the full model, suggesting that exhaustion occurs over longer timescales than the 72-hour experimental window. Parameter estimates are in Table A2.

### 3.2 Monotherapy Analysis

#### 3.2.1 Oncolytic Virus Monotherapy

For oncolytic virus monotherapy, the full model Eq. (5) explicitly tracks viral dynamics through three coupled differential equations:


(11) $$\frac{\text{dU}}{\text{dt}}=\underset{\text{tumor}\;\text{proliferation}}{\underbrace{\text{rU}\left(1-\frac{U+I}{K}\right)}}-\underset{\text{viral}\;\text{infection}}{\underbrace{\frac{\beta \text{UV}}{U+I+a}}},\;\frac{\text{dI}}{\text{dt}}=\underset{\text{new}\;\text{infections}}{\underbrace{\frac{\beta \text{UV}}{U+I+a}}}-\underset{\text{viral}\;\text{lysis}}{\underbrace{w_{I}I}},\;\frac{\text{dV}}{\text{dt}}=\underset{\text{virus}\;\text{production}}{\underbrace{bw_{I}I}}-\underset{\text{infection}\;\text{loss}}{\underbrace{\frac{\beta \text{UV}}{U+I+a}}}-\underset{\text{viral}\;\text{clearance}}{\underbrace{w_{V}V}}.$$

Here U denotes uninfected tumor cells, I infected tumor cells, and V free viral particles. The infection term incorporates Michaelis–Menten saturation kinetics through the parameter a. Viral dynamics are governed by production via lysis at rate $w_{I}$ with burst size b, consumption during infection at rate β, and natural clearance at rate $w_{V}$, allowing mechanistic tracking of each process.

**Fig. 3:**
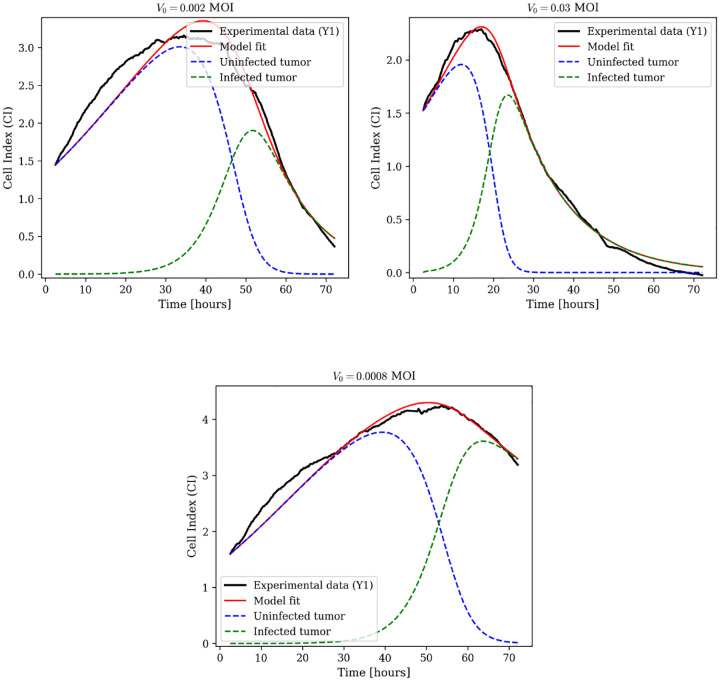
Oncolytic virus monotherapy dynamics using the full model with explicit viral dynamics. Model fits (red lines) to experimental Cell Index data (black lines) for biological replicate Y1 at three MOI levels: 0.002, 0.03, and 0.0008. Dashed blue lines represent uninfected tumor cell dynamics; dashed green lines represent infected tumor cell dynamics. Additional replicates Y2 and Y3 are shown in Appendix A3.

We evaluated oncolytic virus monotherapy at three MOI levels (0.03, 0.002, 0.0008) with three biological replicates per condition. Data-fitting results for biological replicate Y1 are in Fig. 3, with additional replicates (Y2 and Y3) in Appendix A3. The model captures viral oncolysis across three orders of magnitude in viral dose. Parameter estimates are in Table A3.

#### 3.2.2 CAR-T Cell Monotherapy

In the absence of oncolytic virus, the system in Eq. (5) reduces to a two-compartment model describing the interaction between uninfected tumor cells U and CAR-T cells C. The full model incorporating exhaustion dynamics is


(12) $$\frac{\text{dU}}{\text{dt}}=\text{rU}\left(1-\frac{U}{K}\right)-\frac{d_{c}\text{CU}}{U+a},\;\frac{\text{dC}}{\text{dt}}=\frac{\rho \text{CU}}{U+a}-w_{C}C-\frac{\varepsilon \text{CU}}{U+a}.$$

To assess the role of exhaustion over the experimental timescale, we also consider a reduced formulation obtained by setting ε=0, which removes the exhaustion term:


(13) $$\frac{\text{dU}}{\text{dt}}=\text{rU}\left(1-\frac{U}{K}\right)-\frac{d_{c}\text{CU}}{U+a},\;\frac{\text{dC}}{\text{dt}}=\frac{\rho \text{CU}}{U+a}-w_{C}C.$$

Both formulations include logistic tumor growth and CAR-T-mediated killing. CAR-T cell dynamics account for antigen-dependent expansion and natural death, with the full model additionally capturing functional decline due to sustained antigen exposure. The parameter a represents the half-saturation constant governing both killing efficiency and proliferative response.

**Fig. 4:**
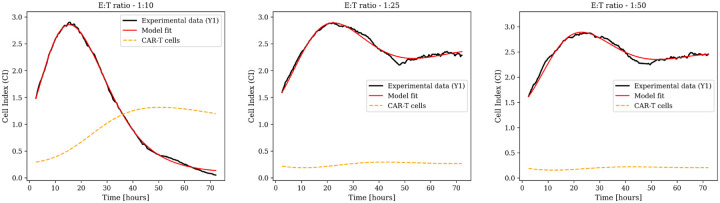
Full model (12) fits for CAR-T cell monotherapy (biological replicate Y1) at three effector-to-target ratios. Solid red lines represent model predictions, black lines indicate experimental Cell Index data, and dashed yellow lines show CAR-T cell dynamics. Parameter estimates are in Table A4. Additional replicates (Y2 and Y3) are shown in Appendix A4.

**Fig. 5:**
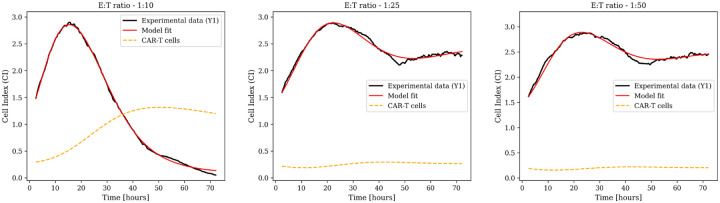
Model without exhaustion (13) fits for CAR-T cell monotherapy (biological replicate Y1) at three effector-to-target ratios. Solid red lines represent model predictions, black lines indicate experimental Cell Index data, and dashed yellow lines show CAR-T cell dynamics. Parameter estimates are in Table A5. Additional replicates (Y2 and Y3) are shown in Appendix A5.

Both formulations were evaluated across three effector-to-target ratios of 1:10, 1:25, and 1:50, corresponding to high, intermediate, and low CAR-T cell concentrations relative to the initial tumor burden. Model fits for biological replicate Y1 are shown in Figs. 4 and 5. Parameter estimates are reported in Tables A4 and A5. Results for additional replicates Y2 and Y3 are provided in Appendices A4 and A5.

### 3.3 Quasi-Steady-State Approximations

Viral replication and clearance occur on faster timescales than cellular processes. Oncolytic viruses exhibit rapid burst kinetics and clearance within hours, while cellular proliferation, death, and phenotypic transitions unfold over days. This separation of timescales allows a quasi-steady-state approximation, where viral populations equilibrate rapidly relative to cellular dynamics.

#### 3.3.1 Combination Therapy Under QSS

Setting $\frac{\text{dV}}{\text{dt}}=0$ in the full model (5) and solving for viral concentration yields:

(14) $$V=\frac{bw_{I}I(U+I+a)}{\beta U+w_{V}(U+I+a)}.$$

When viral clearance dominates reinfection $\left(w_{V}\gg \frac{\beta U}{U+I+a}\right)$, this simplifies to:

(15) $$V\approx \frac{bw_{I}}{w_{V}}I\equiv \alpha I,$$

where $\alpha =\frac{bw_{I}}{w_{V}}$ represents the effective viral amplification factor. Substituting this expression into the full model reduces it to three dimensions:

(16) $$\frac{\text{dU}}{\text{dt}}=\text{rU}\left(1-\frac{U+I}{K}\right)-\frac{\beta \alpha UI}{U+I+a}-\frac{d_{c}\text{CU}}{U+I+a},\;\frac{\text{dI}}{\text{dt}}=\frac{\beta \alpha UI}{U+I+a}-\frac{d_{i}\text{CI}}{U+I+a}-w_{I}I,\;\frac{\text{dC}}{\text{dt}}=\frac{\rho \text{CU}}{U+I+a}-w_{C}C.$$

**Fig. 6:**
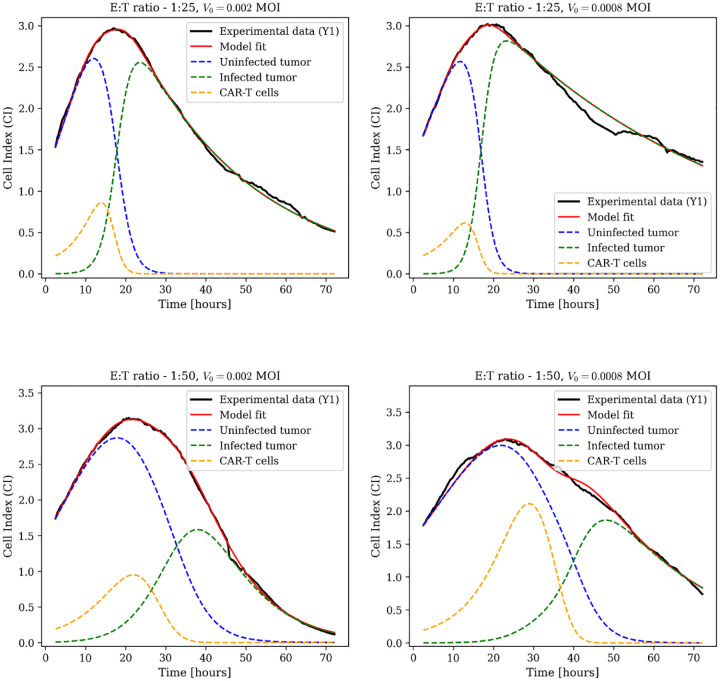
Combination therapy dynamics using the quasi-steady-state model. Model fits (red lines) to experimental Cell Index data (black lines) for biological replicate Y1 across different conditions: E:T ratio 1:25 with MOI 0.002, E:T ratio 1:25 with MOI 0.0008, E:T ratio 1:50 with MOI 0.002, and E:T ratio 1:50 with MOI 0.0008. Dashed blue lines represent uninfected tumor cell dynamics; dashed green lines represent infected tumor cell dynamics; dashed yellow lines represent CAR-T cells. Additional replicates Y2 and Y3 are shown in Appendix A6.

QSS model fits for biological replicate Y1 across varying CAR-T cell doses (E:T ratios of 1:25 and 1:50) and viral loads (MOI of 0.002 and 0.0008) are shown in Fig. 6, with additional replicates (Y2 and Y3) in Appendix A6. Parameter estimates are in Table A6.

#### 3.3.2 Oncolytic Virus Monotherapy Under QSS

For oncolytic virus therapy without CAR-T cells, we set C≡0 in Eq. (16) and remove all CAR-T-related terms. The resulting susceptible-infected system describes uninfected and virus-infected tumor cell dynamics:

(17) $$\frac{\text{dU}}{\text{dt}}=\text{rU}\left(1-\frac{U+I}{K}\right)-\frac{\beta \alpha UI}{U+I+a},\;\frac{\text{dI}}{\text{dt}}=\frac{\beta \alpha UI}{U+I+a}-w_{I}I.$$

**Fig. 7:**
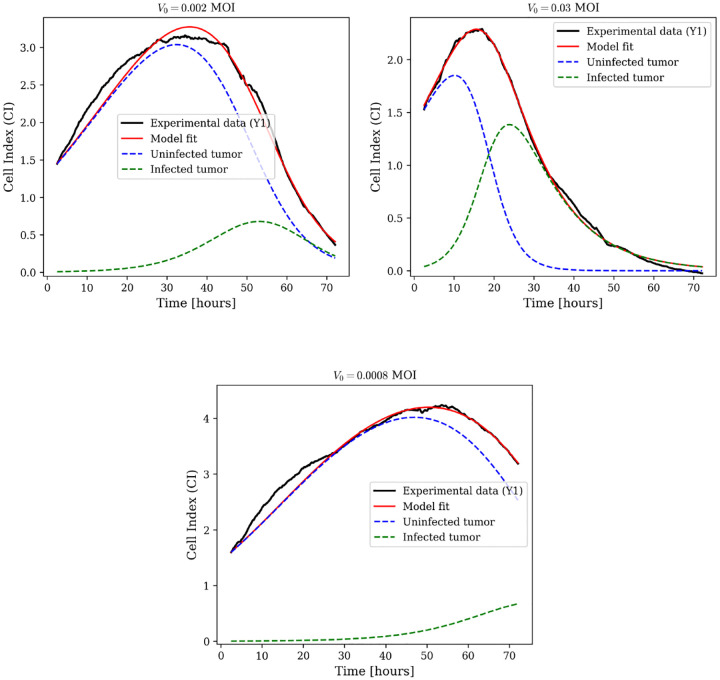
Oncolytic virus monotherapy dynamics using the quasi-steady-state model. Model fits (solid red lines) to experimental Cell Index data (black lines) for biological replicate Y1 at three MOI levels: 0.002, 0.03, and 0.0008. Dashed blue lines represent uninfected tumor cell dynamics; dashed green lines represent infected tumor cell dynamics. Additional replicates Y2 and Y3 are shown in Appendix A7.

QSS model fits for biological replicate Y1 across three MOI levels (0.03, 0.002, 0.0008) are shown in Fig. 7, with additional replicates (Y2 and Y3) in Appendix A7. Parameter estimates are in Table A7.

For CAR-T cell monotherapy, the reduced model without exhaustion (Eq. 13) serves as the quasi-steady-state (QSS) representation. Since this system is identical to the original reduced model, the predicted dynamics and parameter estimates are unchanged.

#### 3.3.3 CAR-T Cell Monotherapy Under QSS

For CAR-T cell monotherapy, the reduced model without exhaustion (Eq. 13) serves as the quasi-steady-state representation. Since no viral dynamics are present, this system is identical to the reduced model, and the predicted dynamics and parameter estimates remain unchanged.

## 4 Results

### 4.1 Performance Metrics for Model Comparison

We compared three model formulations: the Full model (F) with exhaustion dynamics Eq. (5), the model without exhaustion (W) Eq. (10), and the quasi-steady-state approximation (QSS) Eq. (16). These formulations were evaluated across all experimental conditions, including both monotherapy and combination treatment settings.

Table 2 compares two model formulations for oncolytic virus monotherapy across three viral doses. Since no CAR-T cells are present in these experiments, the Full model (F) Eq. (11) is equivalent to the model without exhaustion (W), and both include explicit viral dynamics with six parameters. The QSS approximation uses one fewer parameter Eq. (17) (p=5), and produces fits that are similar to or slightly better than those of the full model Eq. (11) across all MOI levels. At the lowest viral dose (MOI = 0.0008), the QSS model gave RSS = 3.06 ± 1.45 compared to 6.12 ± 1.62 for the full model, with corresponding AIC values of −1223 ± 144 versus −1017 ± 76. At the highest dose (MOI = 0.03), both models fit the data well (adjusted $R^{2}>0.99$), with the full model giving RSS = 1.05 ± 0.27 and AIC = −1486 ± 75, while the QSS model showed slightly lower RSS and AIC (RSS = 0.57 ± 0.37, AIC = −1683 ± 166). These results suggest that the QSS approximation captures the main effects of viral dynamics while simplifying the model.

Table 3 compares model formulations for CAR-T monotherapy across three effector-to-target ratios. In the absence of an oncolytic virus, the model without exhaustion (W) Eq. (13) is equivalent to the QSS approximation. Both the Full model with exhaustion Eq. (12) dynamics and the W=QSS formulation Eq. (13) yielded nearly identical fits across all E:T ratios. At E:T = 1:10, both models gave RSS = 0.354 ± 0.136 and AIC values around −1800, with adjusted $R^{2}=0.998\pm 0.001$. This pattern was similar at E:T = 1:25 (RSS = 0.495 ± 0.296, AIC ≈ −1729) and E:T = 1:50 (RSS ≈ 0.58, AIC ≈ −1640). The exhaustion parameter showed high uncertainty in all conditions (ranging from ϵ=4.679±2.232 at E:T = 1:25 to 6.555 ± 1.419 at E:T = 1:50), and including it did not change the model fit. Overall, the simpler W=QSS formulation with five parameters captures CAR-T monotherapy dynamics within the 72-hour experimental window.

Table 4 compares the Full (F) Eq. (5) and quasi-steady-state (QSS) Eq. (16) model formulations across four combination therapy conditions varying in effector-to-target ratio and viral dose. Both models provided good fits to the data (adjusted $R^{2}>0.98$). The QSS model employs fewer parameters (p=9 versus 11 for F) and generally yields lower RSS and AIC values. At E:T = 1:25 with MOI = 0.002, the QSS model had RSS = 0.486±0.258 and AIC = −1697±133. At the same E:T ratio but lower viral dose (MOI = 0.0008), both models performed similarly. At E:T = 1:50 with MOI = 0.002, the QSS model gave RSS = 0.387 ± 0.150 and AIC = −1832 ± 105. At E:T = 1:50 with MOI = 0.0008, both models performed similarly, with the QSS model slightly better. Including the model without exhaustion (W) in Table A8 shows that W is competitive at E:T = 1:25 with MOI = 0.0008 (RSS = 0.517±0.219, AIC = −1629±116), but overall differences between the three formulations are small. The QSS model provides a good balance of fit and parameter simplicity.

These results show two main trends across the experimental conditions. First, viral dynamics occur on a faster timescale than tumor growth. In OV monotherapy, the QSS formulation Eq. (17) generally fits the data as well as or better than the full model Eq. (17), reducing RSS by roughly two-thirds at intermediate MOI levels while using one fewer parameter. This indicates that the explicit differential equations governing viral production and clearance can be replaced by a quasi-steady-state approximation, assuming rapid equilibration of viral dynamics, without reducing predictive accuracy. Second, exhaustion dynamics do not noticeably affect therapeutic outcomes in this system. In CAR-T monotherapy, models with Eq. (12) and without exhaustion Eq. (13) gave nearly identical fits across all E:T ratios, and including the exhaustion parameter did not improve the model. In combination therapy, these patterns remain: the QSS formulation Eq. (16) performs similarly to the full model Eq. (5) using 9 parameters instead of 11, and the model without exhaustion Eq. (10) gives comparable results (Table A8). Overall, the QSS model reduces model complexity and focuses on the slower dynamics that dominate the experimental timescale while keeping the system interpretable.

### 4.2 Parameter Estimation Across Model Formulations

#### 4.2.1 Oncolytic Virus Monotherapy

We compared the Full model Eq. (5), which includes explicit viral dynamics with six parameters, to the quasi-steady-state (QSS) Eq. (17) approximation with five parameters for OV monotherapy (Table A9). In the absence of CAR-T cells, the Full model is equivalent to the model without exhaustion, as the exhaustion term applies only to CAR-T dynamics; these formulations are denoted F=W in Table A9.

Tumor growth rates were consistent across models and viral doses, ranging from r=0.048 to 0.060 day^−1^. The saturation threshold a converged to approximately 0.10 in both models at low and intermediate MOI, with minimal variability.

The viral infection rate β showed model-dependent behavior. In the Full model Eq. (5),, β ranged from 1.03 to 1.54 and increased with viral dose, consistent with mass-action kinetics. In the QSS model Eq. (17), estimates reached the upper bound of 1.50 at low and intermediate MOI, indicating identifiability issues when viral dynamics are not modeled explicitly. The infected cell lysis rate $w_{I}$ differed between formulations at low and intermediate MOI: the QSS model produced values two- to four-fold higher than the Full model (0.142–0.168 vs. 0.035–0.078 day^−1^). At high MOI, estimates converged (QSS: 0.085, Full: 0.074 day^−1^), reflecting that the QSS formulation combines viral production and clearance into an effective lysis rate, whereas the Full model treats these processes separately.

Parameters unique to the Full model Eq. (5), also exhibited identifiability challenges. The burst size b varied from 13 to 24 virions per cell without a clear dose-dependent trend. The viral clearance rate $w_{V}$ reached its upper bound (~2.0 day^−1^), corresponding to a half-life of ~8 hours, indicating that decay occurs faster than the measurement interval can capture.

In contrast, the QSS parameter α provides a simpler alternative. It increased with viral dose, from 0.178±0.033 at MOI = 0.0008 to 0.746±0.523 at MOI = 0.03, effectively capturing the net oncolytic effect without requiring explicit estimation of fast viral kinetics.

#### 4.2.2 CAR-T Cell Monotherapy

We compared the Full model Eq. (12), which incorporates exhaustion dynamics with six parameters, to the model without exhaustion Eq. (13), which uses five parameters, for CAR-T monotherapy (Table A10). Since no oncolytic virus is present, the quasi-steady-state approximation reduces to the model without exhaustion, and only two formulations were compared for this therapeutic modality.

Tumor growth rates were consistent between formulations, ranging from r=0.227 to 0.254 day^−1^, corresponding to doubling times of 2.7–3.1 days. CAR-T-related parameters displayed dose-dependent trends. The killing rate of uninfected tumor cells, $d_{c}$, decreased from 10.0 day^−1^ at E:T = 1:10 to 8.0 day^−1^ at E:T = 1:50, likely reflecting reduced per-cell cytotoxic efficiency when effectors engage multiple targets sequentially. This accounts for the time required for immunological synapse formation, granule release, and detachment before the next engagement. The saturation threshold a showed a similar pattern, decreasing from 45.2 to 13.8 across the same E:T range, suggesting killing efficiency saturates at lower tumor densities when fewer effectors are available. Conversely, the natural death rate $w_{c}$ increased from 0.016 day^−1^ at E:T = 1:10 to 0.117 day^−1^ at E:T = 1:50, possibly reflecting enhanced CAR-T survival at higher effector densities due to abundant antigen stimulation and autocrine survival signals. These parameters were identical between model formulations.

The proliferation rate ρ differed substantially between models. In the Full model Eq. (12), ρ=5.3−7.3 day^−1^, whereas in the model without exhaustion, ρ=0.62−1.26 day^−1^. This discrepancy arises because the exhaustion term reduces CAR-T function, forcing the optimizer to increase proliferation to match observed tumor killing. Proliferation rates from the model without exhaustion align with known T cell division times of 12–24 hours, whereas Full model Eq. (12) estimates exceed physiological rates. The exhaustion parameter, ϵ=4.7−6.6, showed high uncertainty and did not improve model fit, confirming it cannot be reliably estimated from tumor growth data over this timescale.

#### 4.2.3 Combination Therapy

We compared all three formulations for combination therapy: Full model with exhaustion Eq. (5) using eleven parameters, model without exhaustion Eq. (10) using ten parameters, and quasi-steady-state approximation using nine parameters Eq. (16). Parameter estimates are shown in Table A11.

The tumor growth rate of r=0.086−0.148 day^−1^ and infected cell lysis rate of $w_{i}=0.037-0.085$ day^−1^ were consistent across all three formulations, confirming these parameters can be reliably estimated. The CAR-T proliferation rate of ρ=0.5−1.5 day ^−1^ showed better consistency than in monotherapy, with all estimates in the plausible range for activated T cells.

Other parameters varied considerably across formulations. The CAR-T killing rate of uninfected cells $d_{c}$ differed up to 21-fold within identical conditions. At E:T = 1:50 with MOI = 0.002, the Full model estimated $d_{c}=0.274$ day^−1^, the model without exhaustion returned $d_{c}=1.357$ day^−1^, and the quasi-steady-state model predicted $d_{c}=0.065$ day^−1^. This variability suggests that CAR-T killing effects, including direct cytotoxicity and their interaction with viral dynamics, cannot be separated based on tumor-level data alone. The killing rate of infected cells $d_{i}$ and saturation threshold a showed similar patterns.

The CAR-T natural death rate $w_{c}$ varied across formulations. The quasi-steady-state model consistently gave higher estimates of 0.876–0.976 day^−1^ than the Full model of 0.143–0.497 day^−1^ or the model without exhaustion of 0.137–1.029 day^−1^. The viral infection rate of β=0.33−1.48 also varied without clear dose-dependent trends.

Viral dynamics parameters showed the same identifiability issues seen in monotherapy. The burst size of b=17−52 virions/cell and clearance rate of $w_{V}=0.47-1.24$ day^−1^ varied widely. The exhaustion parameter of ϵ=0.62−1.06 was lower than in CAR-T monotherapy but remained poorly constrained.

The quasi-steady-state parameter α varied across conditions—stable in some with E:T = 1:50, MOI = 0.002: α=0.22±0.01 but variable in others with E:T = 1:25, MOI = 0.002: α=2.04±1.93—suggesting that interactions between viral and CAR-T effects are absorbed differently depending on the therapeutic balance.

Taken together, these results show that the quasi-steady-state formulation achieves comparable or more stable estimates for key parameters of $r,w_{i},\rho$ while avoiding poorly constrained terms of $b,w_{V},\epsilon$, supporting the use of simpler models for reliable biological inference.

## 5 Discussion and Conclusion

Our results demonstrate that minimal model formulations perform comparably to mechanistically detailed alternatives for predicting combination immunotherapy outcomes. The quasi-steady-state (QSS) model Eq. (16) uses nine parameters in combination therapy compared to eleven in the Full model Eq. (5), yet achieves comparable fits. This makes it better suited for applications relying on parameter estimates from tumor data, including treatment optimization, dose scheduling, and outcome prediction.

Incorporating distinct CAR-T exhaustion states did not improve model fit within the 72-hour observation period, reflecting the distinct temporal scales governing T cell dysfunction. Phenotypic exhaustion develops over 2–4 weeks through extensive epigenetic and transcriptional reprogramming [24]. Within a 72-hour window, CAR-T cells have not yet undergone this commitment; instead, functional decline is driven by activation-induced cell death (AICD), which occurs within hours to days. The minimal formulation with effective death rates captured the dominant processes observable within this temporal window without requiring additional exhaustion parameters.

Similar identifiability issues arise for viral dynamics. The viral clearance rate consistently reached parameter bounds, indicating that decay occurs faster than can be resolved by daily measurements. Burst size b estimates varied widely and lacked consistent trends across conditions. The QSS approximation Eq. (17) accounts for this timescale separation by replacing poorly constrained parameters with a single term representing the net oncolytic effect. The monotonic increase of this parameter with viral dose confirms that meaningful therapeutic information is preserved despite the reduced representation.

Predictive performance depends not on mechanistic detail alone, but on whether parameters can be constrained by available data. Phan et al. [25] showed that complex models require more data for parameter identification, while Gevertz and Kareva [26] demonstrated that identifiability analysis can guide both experimental design and model selection. Under data-limited conditions, representing the leading mechanisms supported by observations is more informative than introducing biologically plausible but unresolvable structure. For extended treatment courses beyond 2–4 weeks, models could incorporate reversible and terminal exhaustion states; however, for acute assays, minimal formulations are sufficient.

Comparing model formulations allows separation of robustly identifiable parameters from those sensitive to model structure. The tumor growth rate was the most consistent across all contexts, showing minimal variation, reflecting its clear contribution to observed tumor dynamics during control and early treatment phases. The infected cell lysis rate in combination therapy was also stable across formulations, indicating that virus-mediated cell death can be accurately captured. CAR-T proliferation showed improved consistency in combination therapy relative to monotherapy, suggesting that interacting mechanisms provide additional information to constrain this parameter.

The impact of model complexity on parameter behavior is evident when examining estimates across formulations. The model without exhaustion Eq. (10) produced CAR-T proliferation rates (ρ=0.62−1.26 day^−1^), whereas the Full model Eq. (5) yielded inflated values (ρ=5.3−7.3 day^−1^) that would be difficult to sustain over the 72-hour observation period. This discrepancy reflects non-identifiability between proliferation and exhaustion terms: when exhaustion suppresses CAR-T activity, the optimizer compensates by inflating proliferation rates. The exhaustion parameter itself showed high uncertainty and did not improve fit, confirming it cannot be resolved from tumor data at this timescale.

In contrast to robustly estimated parameters, CAR-T killing rates for both uninfected $d_{c}$ and infected cells $d_{i}$ varied substantially between models, preventing reliable interpretation of direct cytotoxic effects from tumor data alone. Viral burst size b and clearance $w_{V}$ remained non-identifiable wherever explicit kinetics were included. Despite this variability, several parameters exhibited consistent dose-dependent trends: the CAR-T killing rate decreased with increasing tumor burden across all formulations, consistent with limits on consecutive killing, while the QSS parameter α increased with viral dose, matching the expected relationship between viral load and therapeutic impact.

The fact that multiple formulations fit the data equally well highlights a fundamental limitation of inference from aggregate tumor measurements. When several mechanisms contribute to a common outcome, individual effects may be non-identifiable even if the net response is well captured. Processes that proved non-identifiable, including detailed viral kinetics and CAR-T exhaustion, would require complementary data: direct viral titer measurements, flow cytometry of CAR-T phenotypes and exhaustion markers, or single-cell functional assays. Parameter estimates from detailed models should not be over-interpreted as true biological rates when simpler formulations achieve similar accuracy with improved reliability.

Our results highlight that useful models balance mechanistic detail with what the data can reliably resolve. The most informative model is not necessarily the most detailed, but rather one whose structure supports robust parameter estimation and meaningful biological interpretation.

## Supplementary Material

1

## Figures and Tables

**Table 1:** Model parameters for the CAR-T/OV combination therapy model with exhaustion.

Parameter	Biological Interpretation	Units
r	Tumor cell proliferation rate	h^−1^
K	Tumor carrying capacity	CI
β	Viral infection rate	(MOI·h)^−1^
a	Saturation threshold for interactions	CI
$d_{c}$	CAR-T killing rate (uninfected cells)	h^−1^
$d_{i}$	CAR-T killing rate (infected cells)	h^−1^
$w_{I}$	Infected cell lysis rate	h^−1^
b	Viral burst size (virions per lysed cell)	MOI·(CI)^−1^
$w_{V}$	Viral clearance rate	h^−1^
ρ	CAR-T proliferation rate	h^−1^
$w_{C}$	CAR-T natural death rate	h^−1^
ε	CAR-T exhaustion rate	h^−1^

**Table 2.** Performance metrics comparison for oncolytic virus monotherapy across viral doses. Model variants: F=W = Full model (equivalent to model without exhaustion since no CAR-T cells are present) with explicit viral dynamics; QSS = quasi-steady-state approximation. Values represent mean ± standard deviation across three biological replicates.

Metric	MOI = 0.0008		MOI = 0.002		MOI = 0.03	
	F=W	QSS	F=W	QSS	F=W	QSS
AIC	−1017 ± 76	−1223 ± 144	−948 ± 108	−1234 ± 81	−1486 ± 75	−1683 ± 166
RSS	6.12 ± 1.62	3.06 ± 1.45	8.23 ± 2.92	2.83 ± 0.82	1.05 ± 0.27	0.57 ± 0.37
$\text{Adj.}\;R^{2}$	0.950 ± 0.007	0.976 ± 0.010	0.962 ± 0.017	0.987 ± 0.005	0.993 ± 0.002	0.996 ± 0.002
n	270	270	271	271	269	269
p	6	5	6	5	6	5

**Table 3.** Performance metrics comparison for CAR-T cell monotherapy across effector-to-target ratios. Model variants: F = Full model with exhaustion dynamics; W=QSS = model without exhaustion (equivalent to QSS approximation since no viral dynamics are present). Values represent mean ± standard deviation across three biological replicates.

Metric	E:T = 1:10		E:T = 1:25		E:T = 1:50	
	F	W=QSS	F	W=QSS	F	W=QSS
AIC	−1800 ± 102	−1802 ± 102	−1728 ± 184	−1730 ± 184	−1640 ± 68	−1641 ± 68
RSS	0.354 ± 0.136	0.354 ± 0.136	0.495 ± 0.296	0.495 ± 0.296	0.576 ± 0.134	0.579 ± 0.140
$\text{Adj.}\;R^{2}$	0.998 ± 0.001	0.998 ± 0.001	0.980 ± 0.010	0.980 ± 0.010	0.979 ± 0.012	0.979 ± 0.012
n	271	271	270	270	268	268
p	6	5	6	5	6	5

**Table 4.** Performance metrics comparison between Full and QSS model formulations for combination therapy under different experimental conditions. Column headers indicate effector-to-target ratio (E:T) and multiplicity of infection (MOI). Model variants: F = Full model; QSS = quasi-steady-state approximation. Values represent mean ± standard deviation across three biological replicates. Complete comparison including the model without exhaustion is provided in Table A8.

Metric	1:25, 0.002		1:25, 0.0008		1:50, 0.002		1:50, 0.0008	
	F	QSS	F	QSS	F	QSS	F	QSS
AIC	−1640±145	−1697±133	−1539±191	−1505±67	−1694±135	−1832±105	−1441±91	−1500±219
RSS	0.599±0.326	0.486±0.258	0.784±0.428	0.802±0.216	0.638±0.267	0.387±0.150	1.104±0.390	1.090±0.914
$\text{Adj.}\;R^{2}$	0.997±0.002	0.997±0.001	0.994±0.003	0.992±0.006	0.997±0.002	0.998±0.001	0.989±0.005	0.992±0.004
n	268	268	262	262	279	279	265	265
p	11	9	11	9	11	9	11	9

## Data Availability

The data used for this study are openly available at https://github.com/mconte93/CAR-OV-study.git.
