# Supplementary material for: A Minimal Model Framework for Robust CAR-T Cell and Oncolytic Virus Combination Therapy
Source: Res Sq. 2026 Jan 27:rs.3.rs-8680401. Preprint. [Version 1] doi: 10.21203/rs.3.rs-8680401/v1 (PMC12869583; doi:10.21203/rs.3.rs-8680401/v1)
Supplement: 1 [file NIHPPRS8680401V1-supplement-1.pdf]

Appendix A Supplementary Figures

This appendix provides complete data for all three biological replicates (Y1, Y2, and Y3) for all experimental conditions presented in the main text. The main text figures display only the first biological replicate (Y1) for clarity. This appendix provides additional figures for replicates Y2 and Y3, as well as tables showing parameter estimates and performance metrics across all three replicates (Y1, Y2, Y3), demonstrating the consistency and reproducibility of the model fits.

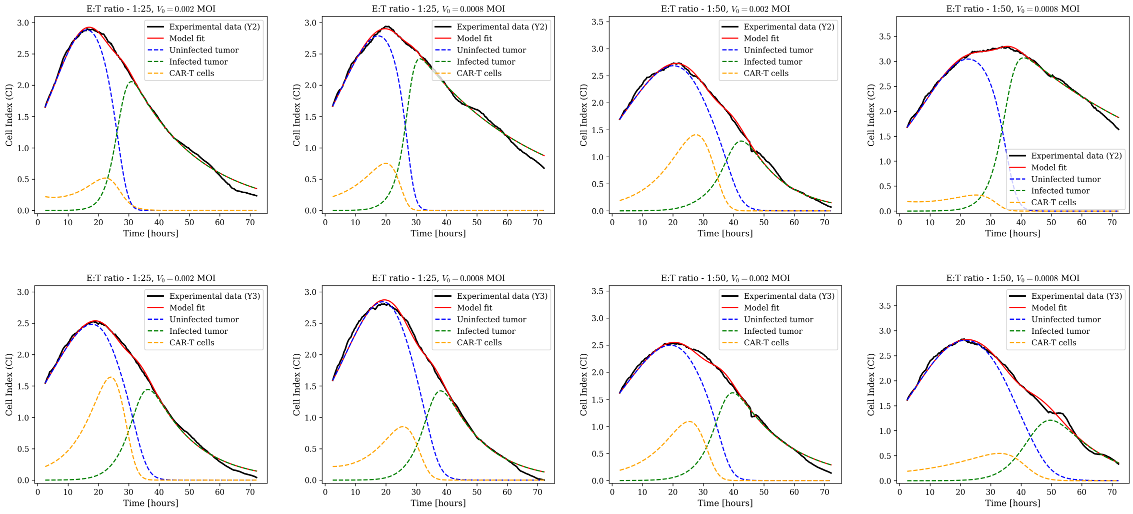

Fig. A1: Additional biological replicates (Y2 and Y3) for combination therapy dynamics using the full model with CAR-T cell exhaustion. Model fits (red lines) to experimental Cell Index data (black lines). Dashed blue lines represent uninfected tumor cell dynamics; dashed green lines represent infected tumor cell dynamics; dashed yellow lines represent CAR-T cells. The top row shows Y2 replicates, the bottom row shows Y3 replicates, across all experimental conditions.

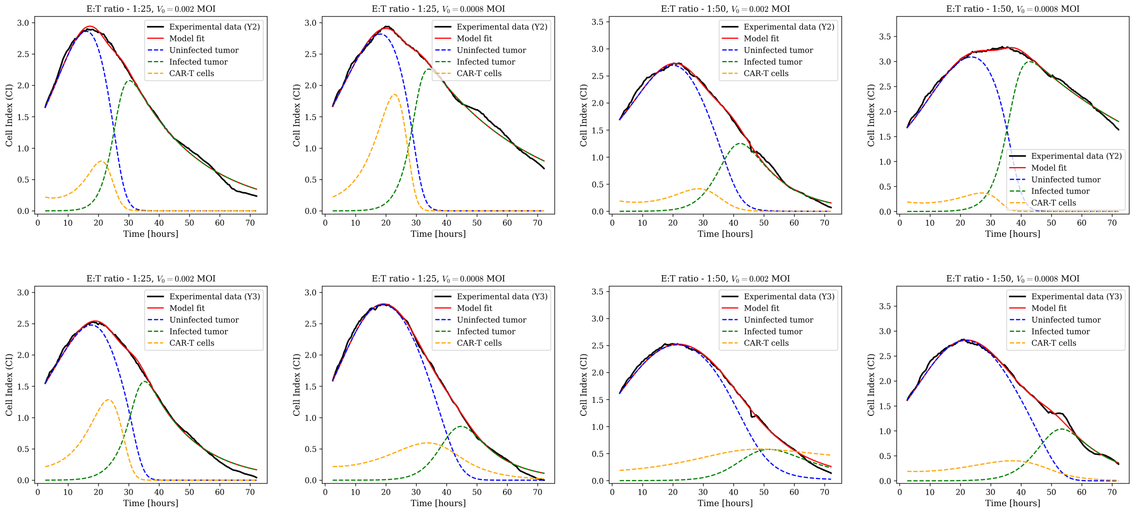

Fig. A2: Additional biological replicates (Y2 and Y3) for combination therapy dynamics using the reduced model without CAR-T cell exhaustion. Model fits (red lines) to experimental Cell Index data (black lines). Dashed blue lines represent uninfected tumor cell dynamics; dashed green lines represent infected tumor cell dynamics; dashed yellow lines represent CAR-T cells. The top row shows Y2 replicates, the bottom row shows Y3 replicates, across all experimental conditions.

Table A1: Parameter estimates and performance metrics for combination therapy in our full model.

Parameter	E:T=1:25, MOI=0.002			E:T=1:25, MOI=0.0008			E:T=1:50, MOI=0.002			E:T=1:50, MOI=0.0008		
	Y1	Y2	Y3	Y1	Y2	Y3	Y1	Y2	Y3	Y1	Y2	Y3
Model Parameters												
r	0.149	0.171	0.091	0.159	0.114	0.128	0.137	0.084	0.084	0.099	0.132	0.153
β	0.484	0.295	0.963	1.236	1.111	0.507	0.837	0.431	0.661	1.024	1.308	0.544
d_c	0.257	1.416	0.101	1.327	0.150	0.326	0.588	0.103	0.131	0.129	0.522	0.579
w_i	0.034	0.044	0.069	0.013	0.025	0.075	0.081	0.080	0.057	0.028	0.017	0.068
w_c	0.044	0.297	0.332	0.092	0.816	0.582	0.144	0.120	0.166	0.038	0.544	0.025
ρ	1.230	1.310	1.387	1.207	1.498	1.391	1.146	1.434	1.452	1.451	1.216	0.679
d_i	0.100	0.214	0.110	1.026	0.710	0.526	0.587	0.249	0.191	0.396	0.559	0.259
a	1.481	4.593	0.302	5.642	0.105	0.741	1.791	0.169	0.370	0.266	0.635	0.570
b	62.648	58.508	17.270	64.683	55.876	33.402	16.156	26.063	18.473	40.041	51.654	20.862
w_v	0.449	0.106	1.473	0.202	1.389	1.352	0.920	1.416	0.840	1.264	1.252	1.195
ϵ	0.964	0.323	0.878	0.777	0.556	0.539	0.824	1.200	1.149	1.311	0.494	0.598
Performance Metrics												
Error	0.015	0.027	0.025	0.011	0.024	0.032	0.016	0.024	0.026	0.024	0.015	0.024
RSS	0.327	0.960	0.509	0.291	1.063	0.999	0.345	0.700	0.869	1.539	0.986	0.786
AIC	-1776	-1487	-1657	-1760	-1421	-1437	-1846	-1649	-1588	-1342	-1461	-1520
Adj. R^2	0.998	0.995	0.997	0.996	0.991	0.996	0.999	0.997	0.995	0.988	0.985	0.995
n (points)	268	268	268	262	262	262	279	279	279	265	265	265
p (params)	11	11	11	11	11	11	11	11	11	11	11	11

Table A2: Parameter estimates and performance metrics for combination therapy in our model without exhaustion term.

Parameter	E:T=1:25, MOI=0.002			E:T=1:25, MOI=0.0008			E:T=1:50, MOI=0.002			E:T=1:50, MOI=0.0008		
	Y1	Y2	Y3	Y1	Y2	Y3	Y1	Y2	Y3	Y1	Y2	Y3
Model Parameters												
r	0.168	0.131	0.093	0.171	0.095	0.177	0.129	0.118	0.132	0.096	0.110	0.178
β	1.326	0.900	0.790	0.726	0.915	0.254	0.125	1.035	0.755	0.745	0.556	0.173
d_c	0.313	0.396	0.122	1.488	0.070	1.500	1.339	1.251	1.482	0.175	0.370	1.500
w_I	0.034	0.045	0.064	0.012	0.029	0.082	0.078	0.080	0.042	0.028	0.019	0.066
w_C	1.085	0.825	1.178	0.081	1.191	0.104	0.010	0.389	0.013	1.041	1.064	0.142
ρ	1.421	1.474	1.456	0.381	1.394	0.371	0.245	1.141	0.130	1.397	1.318	0.325
d_i	0.202	0.244	0.178	0.210	0.154	0.166	0.950	0.580	0.256	0.125	0.364	0.137
a	0.447	1.512	0.287	5.499	0.142	4.025	8.951	4.008	4.639	0.620	0.493	2.329
b	43.587	36.094	14.021	95.311	44.044	17.715	9.869	12.072	29.242	53.551	60.706	15.629
w_V	1.460	1.075	0.617	0.073	1.295	0.043	1.344	0.463	0.799	1.362	0.805	0.013
Performance Metrics												
Error	0.015	0.029	0.028	0.011	0.020	0.021	0.012	0.025	0.021	0.027	0.016	0.022
RSS	0.323	1.034	0.716	0.306	0.743	0.501	0.352	0.746	0.558	1.715	0.739	0.733
AIC	−1782	−1469	−1568	−1749	−1517	−1620	−1843	−1633	−1714	−1316	−1539	−1541
Adj. R^2	0.998	0.995	0.996	0.996	0.994	0.998	0.999	0.997	0.997	0.986	0.989	0.996
n (points)	268	268	268	262	262	262	279	279	279	265	265	265
p (params)	10	10	10	10	10	10	10	10	10	10	10	10

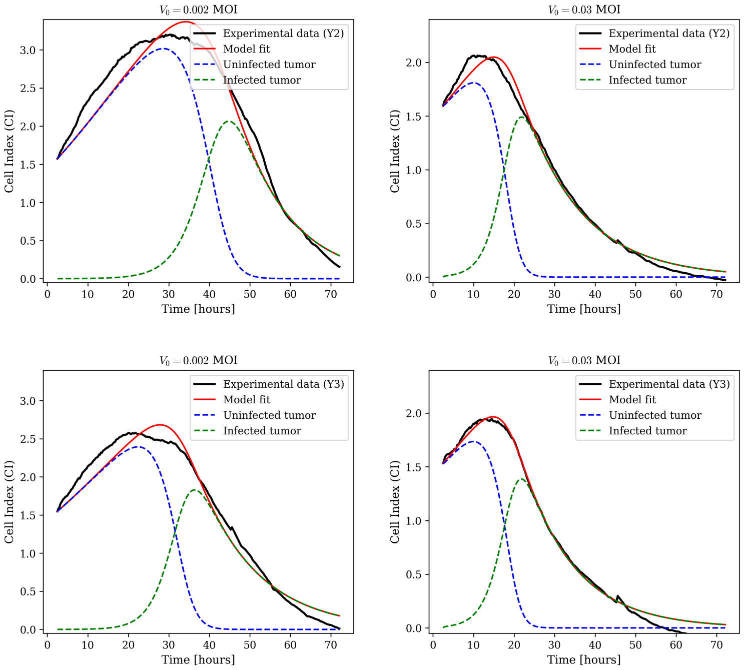

Fig. A3: Additional biological replicates (Y2 and Y3) for oncolytic virus monotherapy dynamics using the full model with explicit viral dynamics. Model fits (red lines) to experimental Cell Index data (black lines). Dashed blue lines represent uninfected tumor cell dynamics; dashed green lines represent infected tumor cell dynamics. The top row shows Y2 replicates, the bottom row shows Y3 replicates, at three MOI levels.

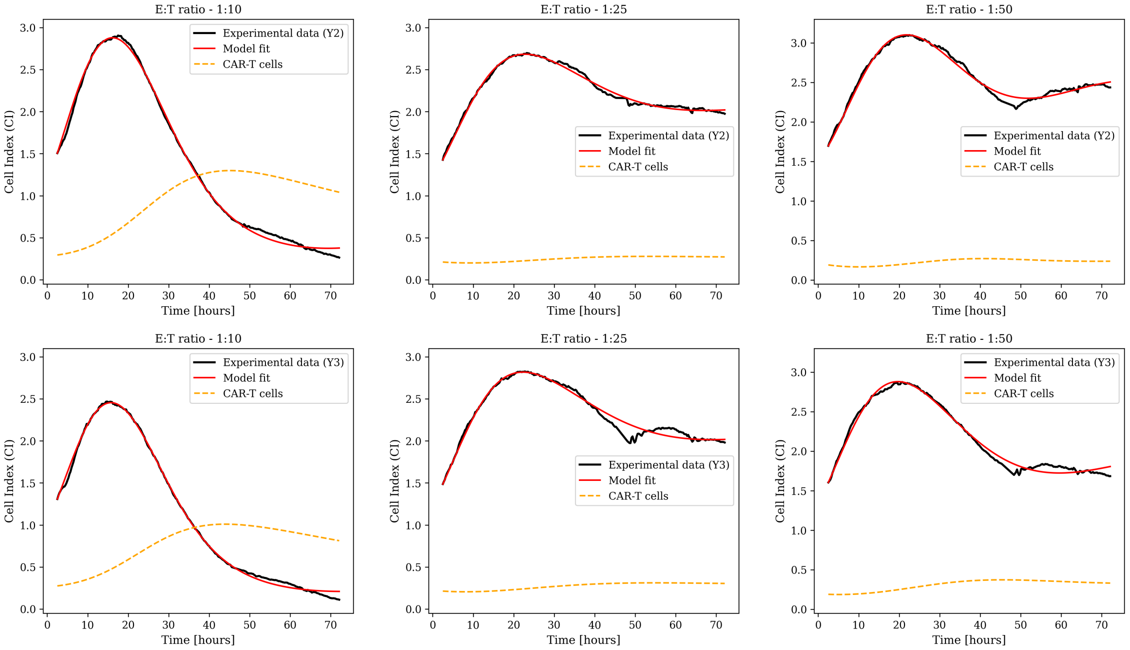

Fig. A4: Additional biological replicates (Y2 and Y3) for CAR-T cell monotherapy using the full model (12). Solid red lines show model fits to experimental Cell Index data (black lines). The top row corresponds to Y2 replicates, and the bottom row corresponds to Y3 replicates, each at three E:T ratios.

Table A3: Parameter estimates for oncolytic virus therapy in the full model without CAR-T cell exhaustion and with explicit viral dynamics.

Parameter	MOI = 0.002			MOI = 0.03			MOI = 0.0008		
	Y1	Y2	Y3	Y1	Y2	Y3	Y1	Y2	Y3
Model Parameters									
r	0.0494	0.0534	0.0429	0.0606	0.0417	0.0420	0.0562	0.0488	0.0463
β	1.1522	1.1398	0.9957	1.4661	1.4038	1.7565	1.0610	0.9408	1.0901
w_I	0.0832	0.0794	0.0698	0.0733	0.0699	0.0786	0.0177	0.0478	0.0399
b	9.8595	11.7211	17.5191	14.1556	16.2676	12.3824	33.4971	19.6918	18.8607
w_V	1.9994	1.9990	2.0000	1.9984	1.9996	1.9640	1.7288	1.9974	1.9974
a	0.1003	0.1008	0.1002	0.1001	0.1008	0.1458	0.1009	0.1002	0.1007
Performance Metrics									
Error	0.0723	0.0717	0.0791	0.0447	0.0633	0.0642	0.0319	0.0542	0.0369
RSS	10.7639	8.9269	5.0036	0.7449	1.1600	1.2541	6.4184	7.5873	4.3535
AIC	−862	−913	−1070	−1572	−1453	−1432	−998	−952	−1102
Adj. R^2	0.943	0.967	0.977	0.996	0.992	0.992	0.950	0.944	0.957
n (points)	271	271	271	269	269	269	270	270	270
p (params)	6	6	6	6	6	6	6	6	6

Table A4: Parameter estimates and performance metrics for the full CAR-T cell monotherapy model (12) across three biological replicates (Y1–Y3) and three effector-to-target (E:T) ratios.

Parameter	E:T = 1:10			E:T = 1:25			E:T = 1:50		
	Y1	Y2	Y3	Y1	Y2	Y3	Y1	Y2	Y3
Model Parameters									
r	0.240	0.216	0.224	0.224	0.281	0.258	0.247	0.240	0.218
w_c	0.010	0.022	0.017	0.119	0.055	0.054	0.131	0.155	0.065
ρ	9.521	5.158	4.174	4.968	2.997	7.934	5.849	8.368	7.618
a	44.953	53.217	37.395	16.386	13.892	17.293	13.169	5.738	22.434
d_c	10.000	10.000	10.000	8.529	10.000	10.000	9.794	4.225	10.000
ϵ	8.427	3.594	3.050	4.021	2.579	7.438	5.017	7.854	6.794
Performance Metrics									
Error	0.017	0.023	0.021	0.013	0.010	0.015	0.015	0.012	0.018
RSS	0.243	0.504	0.315	0.478	0.208	0.799	0.641	0.420	0.666
AIC	−1890	−1692	−1819	−1699	−1924	−1560	−1605	−1719	−1595
Adj. R^2	0.999	0.998	0.998	0.978	0.991	0.972	0.965	0.985	0.986
n (points)	271	271	271	270	270	270	268	268	268
p (params)	6	6	6	6	6	6	6	6	6

Table A5: Parameter estimates and performance metrics for the CAR-T cell monotherapy model without exhaustion (13), across three biological replicates (Y1–Y3) and three effector-to-target (E:T) ratios.

Parameter	E:T = 1:10			E:T = 1:25			E:T = 1:50		
	Y1	Y2	Y3	Y1	Y2	Y3	Y1	Y2	Y3
Model Parameters									
r	0.240	0.216	0.224	0.224	0.281	0.258	0.247	0.240	0.218
w_c	0.010	0.022	0.017	0.119	0.055	0.054	0.129	0.155	0.065
ρ	1.094	1.565	1.124	0.949	0.419	0.496	0.839	0.515	0.824
a	44.954	53.218	37.394	16.441	13.891	17.295	13.445	5.738	22.436
d_c	10.000	10.000	10.000	8.550	10.000	10.000	9.999	4.225	10.000
Performance Metrics									
Error	0.017	0.023	0.021	0.013	0.010	0.015	0.015	0.012	0.018
RSS	0.243	0.504	0.315	0.478	0.208	0.799	0.651	0.420	0.666
AIC	−1892	−1694	−1821	−1701	−1926	−1562	−1604	−1721	−1597
Adj. R^2	0.999	0.998	0.998	0.978	0.991	0.972	0.965	0.985	0.986
n (points)	271	271	271	270	270	270	268	268	268
p (params)	5	5	5	5	5	5	5	5	5

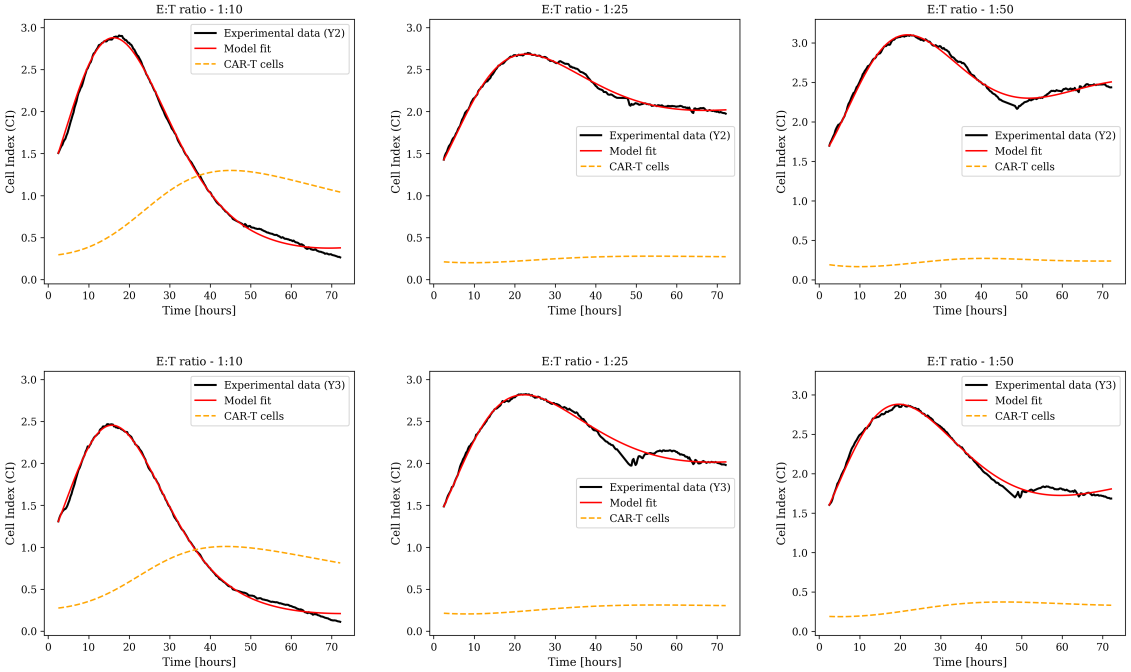

Fig. A5: Additional biological replicates (Y2 and Y3) for CAR-T cell monotherapy using the model without exhaustion (13). Solid red lines show model fits to experimental Cell Index data (black lines). The top row corresponds to Y2 replicates, and the bottom row corresponds to Y3 replicates, each at three E:T ratios.

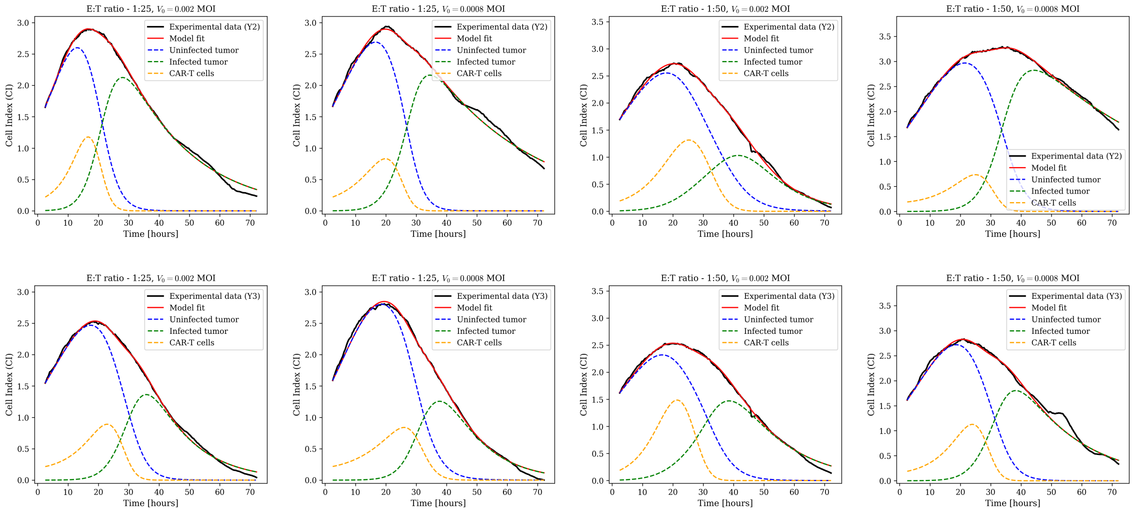

Fig. A6: Additional biological replicates (Y2 and Y3) for combination therapy dynamics using the quasi-steady-state model. Model fits (red lines) to experimental Cell Index data (black lines). Dashed blue lines represent uninfected tumor cell dynamics; dashed green lines represent infected tumor cell dynamics; dashed yellow lines represent CAR-T cells. The top row shows Y2 replicates, the bottom row shows Y3 replicates, across all experimental conditions.

Table A6: Parameter estimates and performance metrics for combination therapy in our QSS model.

Parameter	E:T=1:25, MOI=0.002			E:T=1:25, MOI=0.0008			E:T=1:50, MOI=0.002			E:T=1:50, MOI=0.0008		
	Y1	Y2	Y3	Y1	Y2	Y3	Y1	Y2	Y3	Y1	Y2	Y3
Model Parameters												
r	0.155	0.123	0.105	0.137	0.101	0.143	0.104	0.083	0.072	0.079	0.093	0.097
β	0.388	1.244	0.115	0.503	0.830	0.151	1.490	1.465	1.481	0.283	0.347	0.349
d_c	0.154	0.093	0.184	0.164	0.097	0.304	0.085	0.072	0.039	0.052	0.127	0.119
w_i	0.034	0.045	0.073	0.016	0.029	0.085	0.093	0.100	0.062	0.041	0.019	0.050
w_c	1.169	0.791	0.841	1.283	0.832	0.514	0.904	0.716	1.038	1.120	0.924	0.885
ρ	1.434	1.054	1.035	1.487	0.977	0.634	1.068	0.886	1.259	1.284	1.110	1.076
d_i	0.106	0.108	0.203	0.109	0.407	0.110	0.112	0.105	0.104	0.153	0.104	0.148
a	0.184	0.247	0.254	0.129	0.116	0.215	0.107	0.136	0.105	0.104	0.267	0.208
α	1.591	0.369	4.150	1.329	0.545	2.972	0.224	0.221	0.213	1.200	0.968	1.173
Performance Metrics												
Error	0.015	0.023	0.021	0.019	0.020	0.024	0.012	0.017	0.019	0.017	0.011	0.032
RSS	0.301	0.781	0.377	1.047	0.720	0.639	0.263	0.343	0.554	0.712	0.425	2.132
AIC	−1802	−1547	−1742	−1429	−1527	−1558	−1926	−1852	−1718	−1551	−1688	−1260
Adj. R^2	0.998	0.996	0.998	0.986	0.994	0.997	0.999	0.998	0.997	0.994	0.994	0.987
n (points)	268	268	268	262	262	262	279	279	279	265	265	265
p (params)	9	9	9	9	9	9	9	9	9	9	9	9

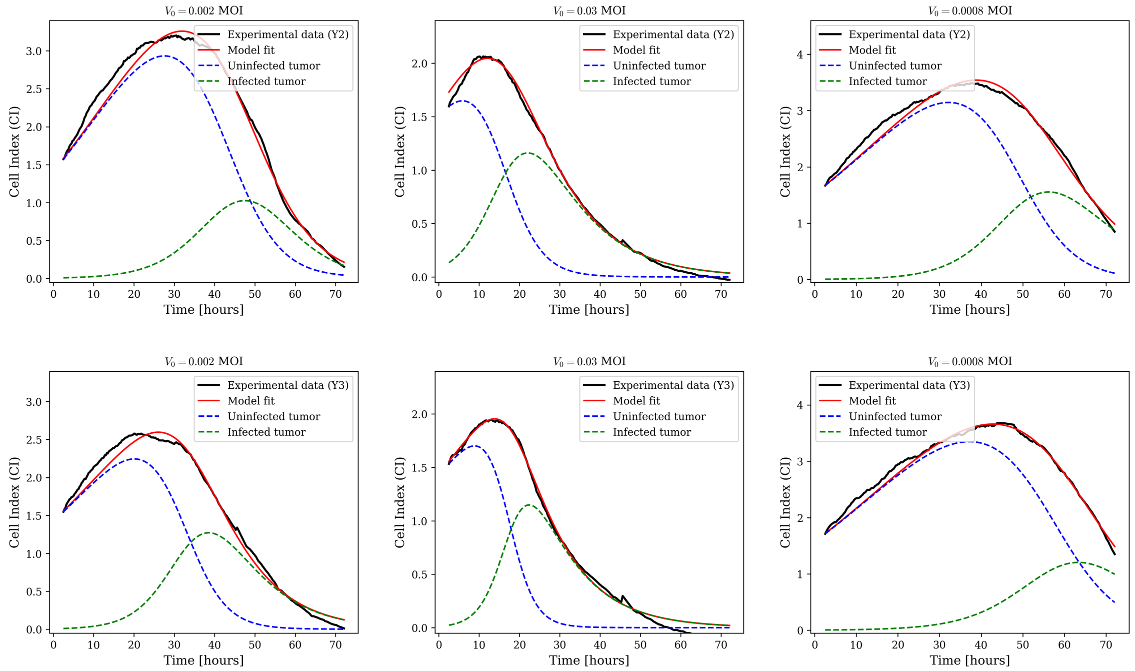

Fig. A7: Additional biological replicates (Y2 and Y3) for oncolytic virus monotherapy dynamics using the quasi-steady-state model. Model fits (red lines) to experimental Cell Index data (black lines). Dashed blue lines represent uninfected tumor cell dynamics; dashed green lines represent infected tumor cell dynamics. The top row shows Y2 replicates, the bottom row shows Y3 replicates, at three MOI levels.

Table A7: Parameter estimates and performance metrics for oncolytic virus monotherapy using the quasi–steady-state (QSS) model.

Parameter	MOI=0.0008			MOI=0.002			MOI=0.03		
	Y1	Y2	Y3	Y1	Y2	Y3	Y1	Y2	Y3
Model Parameters									
α	0.216	0.156	0.162	0.256	0.208	0.195	0.750	0.224	1.264
β	1.497	1.500	1.500	1.500	1.500	1.500	0.505	1.394	0.359
w_I	0.224	0.085	0.118	0.251	0.160	0.092	0.085	0.082	0.089
a	0.100	0.100	0.100	0.100	0.100	0.100	0.148	0.110	0.300
r	0.060	0.053	0.050	0.062	0.063	0.049	0.073	0.060	0.046
Performance Metrics									
Error	0.025	0.032	0.020	0.041	0.037	0.047	0.027	0.033	0.059
RSS	4.465	3.143	1.569	3.667	2.788	2.022	0.305	0.404	0.997
AIC	−1098	−1192	−1380	−1156	−1230	−1317	−1815	−1739	−1496
Adj. R^2	0.965	0.977	0.985	0.981	0.990	0.991	0.998	0.997	0.994
n (points)	270	270	270	271	271	271	269	269	269
p (params)	5	5	5	5	5	5	5	5	5

Table A8: Complete performance metrics comparison across three model formulations for combination therapy under different experimental conditions. Column headers indicate effector-to-target ratio (E:T) and multiplicity of infection (MOI). Model variants: F = Full model with exhaustion dynamics; W = model without exhaustion dynamics; QSS = quasi-steady-state approximation. Values represent mean ± standard deviation across three biological replicates.

	**1:25, 0.002**			**1:25, 0.0008**			**1:50, 0.002**			**1:50, 0.0008**		
Metric	F	W	QSS	F	W	QSS	F	W	QSS	F	W	QSS
RSS	0.599 ±0.326	0.691 ±0.356	0.486 ±0.258	0.784 ±0.428	0.517 ±0.219	0.802 ±0.216	0.638 ±0.267	0.552 ±0.197	0.387 ±0.150	1.104 ±0.390	1.062 ±0.565	1.090 ±0.914
AIC	-1640 ±145	-1606 ±160	-1697 ±133	-1539 ±191	-1629 ±116	-1505 ±67	-1694 ±135	-1730 ±106	-1832 ±105	-1441 ±91	-1465 ±129	-1500 ±219
Adj. R^2	0.997 ±0.002	0.996 ±0.002	0.997 ±0.001	0.994 ±0.003	0.996 ±0.002	0.992 ±0.006	0.997 ±0.002	0.998 ±0.001	0.998 ±0.001	0.989 ±0.005	0.990 ±0.005	0.992 ±0.004
n	268	268	268	262	262	262	279	279	279	265	265	265
p	11	10	9	11	10	9	11	10	9	11	10	9

Table A9: Parameter estimates and performance metrics for oncolytic virus monotherapy. Model variants: F=W = Full model (equivalent to model without exhaustion since no CAR-T cells are present) with explicit viral dynamics ($p = 6$); QSS = quasi-steady-state approximation ($p = 5$). Values represent mean ± standard deviation across three biological replicates (Y1–Y3).

	**MOI = 0.0008**		**MOI = 0.002**		**MOI = 0.03**	
Parameter	F=W	QSS	F=W	QSS	F=W	QSS
r	0.050 ± 0.005	0.054 ± 0.005	0.049 ± 0.005	0.058 ± 0.008	0.048 ± 0.011	0.060 ± 0.014
β	1.030 ± 0.080	1.499 ± 0.002	1.096 ± 0.088	1.500 ± 0.000	1.539 ± 0.184	0.753 ± 0.541
w_I	0.035 ± 0.015	0.142 ± 0.071	0.078 ± 0.007	0.168 ± 0.080	0.074 ± 0.005	0.085 ± 0.004
a	0.101 ± 0.000	0.100 ± 0.000	0.100 ± 0.000	0.100 ± 0.000	0.116 ± 0.025	0.186 ± 0.099
b	24.02 ± 7.78	—	13.03 ± 4.12	—	14.27 ± 1.98	—
w_V	1.975 ± 0.155	—	2.000 ± 0.001	—	1.977 ± 0.020	—
α	—	0.178 ± 0.033	—	0.220 ± 0.032	—	0.746 ± 0.523
RSS	6.12 ± 1.62	3.06 ± 1.45	8.23 ± 2.92	2.83 ± 0.82	1.05 ± 0.27	0.57 ± 0.37
AIC	−1017 ± 76	−1223 ± 144	−948 ± 108	−1234 ± 81	−1486 ± 75	−1683 ± 166
Adj. R^2	0.950 ± 0.007	0.976 ± 0.010	0.962 ± 0.017	0.987 ± 0.005	0.993 ± 0.002	0.996 ± 0.002
n	270	270	271	271	269	269
p	6	5	6	5	6	5

Table A10: Parameter estimates and performance metrics for CAR-T cell monotherapy. Model variants: F = Full model with exhaustion dynamics ($p = 6$); W = model without exhaustion dynamics ($p = 5$). Values represent mean ± standard deviation across three biological replicates (Y1–Y3).

	**E:T = 1:10**		**E:T = 1:25**		**E:T = 1:50**	
Parameter	F	W	F	W	F	W
r	0.227 ± 0.012	0.227 ± 0.012	0.254 ± 0.029	0.254 ± 0.029	0.235 ± 0.015	0.235 ± 0.015
w_c	0.016 ± 0.006	0.016 ± 0.006	0.076 ± 0.037	0.076 ± 0.037	0.117 ± 0.046	0.116 ± 0.046
ρ	6.284 ± 2.805	1.261 ± 0.226	5.300 ± 2.471	0.621 ± 0.270	7.278 ± 1.273	0.726 ± 0.165
a	45.188 ± 7.921	45.189 ± 7.922	15.857 ± 1.743	15.876 ± 1.750	13.780 ± 8.368	13.873 ± 8.369
d_c	10.000 ± 0.000	10.000 ± 0.000	9.510 ± 0.850	9.517 ± 0.855	8.006 ± 3.329	8.075 ± 3.392
ϵ	5.024 ± 2.860	—	4.679 ± 2.232	—	6.555 ± 1.419	—
RSS	0.354 ± 0.136	0.354 ± 0.136	0.495 ± 0.296	0.495 ± 0.296	0.576 ± 0.134	0.579 ± 0.140
AIC	−1800 ± 102	−1802 ± 102	−1728 ± 184	−1730 ± 184	−1640 ± 68	−1641 ± 68
Adj. R^2	0.998 ± 0.001	0.998 ± 0.001	0.980 ± 0.010	0.980 ± 0.010	0.979 ± 0.012	0.979 ± 0.012
n	271	271	270	270	268	268
p	6	5	6	5	6	5

Table A11: Parameter comparison across three model formulations for combination therapy. Model variants: F = Full model with exhaustion dynamics; W = model without exhaustion dynamics; QSS = quasi-steady-state approximation. The Full model contains 11 fitted parameters, the model without exhaustion contains 10 parameters, and the QSS model contains 9 parameters. Values represent mean ± standard deviation across three biological replicates (Y1, Y2, Y3).

	**E:T=1:25, MOI=0.002**			**E:T=1:25, MOI=0.0008**			**E:T=1:50, MOI=0.002**			**E:T=1:50, MOI=0.0008**		
**Param.**	F	W	QSS	F	W	QSS	F	W	QSS	F	W	QSS
Growth & Killing												
r	0.137	0.131	0.128	0.134	0.148	0.127	0.102	0.126	0.086	0.128	0.128	0.090
	±0.041	±0.038	±0.025	±0.023	±0.046	±0.023	±0.031	±0.007	±0.016	±0.027	±0.044	±0.009
β	0.581	1.005	0.582	0.951	0.632	0.495	0.643	0.638	1.479	0.959	0.491	0.326
	±0.344	±0.283	±0.589	±0.390	±0.340	±0.340	±0.204	±0.466	±0.013	±0.386	±0.291	±0.038
d_c	0.591	0.277	0.144	0.601	1.019	0.188	0.274	1.357	0.065	0.410	0.682	0.099
	±0.718	±0.141	±0.046	±0.635	±0.822	±0.106	±0.272	±0.117	±0.024	±0.245	±0.715	±0.041
Infected Cell Dynamics												
w_i	0.049	0.048	0.051	0.038	0.041	0.043	0.073	0.067	0.085	0.038	0.038	0.037
	±0.018	±0.015	±0.020	±0.033	±0.037	±0.037	±0.014	±0.021	±0.020	±0.027	±0.025	±0.016
w_c	0.224	1.029	0.934	0.497	0.459	0.876	0.143	0.137	0.886	0.202	0.749	0.976
	±0.157	±0.183	±0.205	±0.369	±0.634	±0.386	±0.023	±0.218	±0.162	±0.296	±0.526	±0.126
ρ	1.309	1.450	1.174	1.365	0.715	1.033	1.344	0.505	1.071	1.115	1.013	1.157
	±0.079	±0.027	±0.225	±0.147	±0.588	±0.429	±0.172	±0.553	±0.187	±0.396	±0.597	±0.112
d_i	0.141	0.208	0.139	0.754	0.177	0.209	0.342	0.595	0.107	0.405	0.209	0.135
	±0.063	±0.033	±0.055	±0.253	±0.029	±0.172	±0.214	±0.347	±0.004	±0.150	±0.135	±0.027
CAR-T & Viral												
a	2.13	0.75	0.23	2.16	3.22	0.15	0.78	5.87	0.12	0.49	1.15	0.19
	±2.22	±0.67	±0.04	±3.03	±2.77	±0.05	±0.88	±2.69	±0.02	±0.20	±1.03	±0.08
b	46.1	31.2	—	51.3	52.4	—	20.2	17.1	—	37.5	43.3	—
	±25.1	±15.4		±16.1	±39.5		±5.2	±10.6		±15.6	±24.2	
w_v	0.68	1.05	—	0.98	0.47	—	1.06	0.87	—	1.24	0.73	—
	±0.71	±0.42		±0.68	±0.71		±0.31	±0.45		±0.04	±0.68	
ϵ	0.72	—	—	0.62	—	—	1.06	—	—	0.80	—	—
	±0.35			±0.13			±0.20			±0.45		
α	—	—	2.04	—	—	1.62	—	—	0.22	—	—	1.11
			±1.93			±1.24			±0.01			±0.13
